# 963. External Validation of the 2023 Duke - International Society for Cardiovascular Infectious Diseases (ISCVID) Diagnostic Criteria for Infective Endocarditis (IE)

**DOI:** 10.1093/ofid/ofad500.024

**Published:** 2023-11-27

**Authors:** Thomas W van der Vaart, Patrick M M Bossuyt, Larry M Baddour, Arnold S Bayer, David T Durack, Emanuele Durante Mangoni, Thomas L Holland, Adolf W Karchmer, José M Miró, Philippe Moreillon, Magnus Rasmussen, Christine Selton-Suty, Vance G Fowler, Jan T M van der Meer

**Affiliations:** Academic Medical Center Amsterdam, Amsterdam, Noord-Holland, Netherlands; Amsterdam University Medical Center, Amsterdam, Noord-Holland, Netherlands; Mayo Clinic College of Medicine, Rochester, MN; Lundquist Institute-Harbor UCLA Medical Center, Torrance, California; Duke University, Durham, North Carolina; University of Campania 'Luigi Vanvitelli' - Monaldi Hospital, Napoli, Campania, Italy; Duke University Medical Center, Durham, NC; Beth Israel Deaconess Medical Center, Harvard Medical School, Boston, Massachusetts; Hospital Clinic-IDIBAPS, University of Barcelona, Barcelona, Catalonia, Spain; UNIL - Université de Lausanne, Lausanne, Vaud, Switzerland; Lund university; University Hospital of Nancy, Nancy, Lorraine, France; Duke University Medical Center, Durham, NC; Amsterdam University Medical Center, Amsterdam, Noord-Holland, Netherlands

## Abstract

**Background:**

The 2023 Duke-ISCVID criteria for IE were recently introduced to improve classification of IE for research and clinical purposes (Table 1). We compared the diagnostic accuracy of the new criteria with the 2000 modified Duke criteria, the 2015 European Society of Cardiology (ESC) criteria and a clinically adjudicated reference standard by a panel of international experts on IE.

**Table 1:**
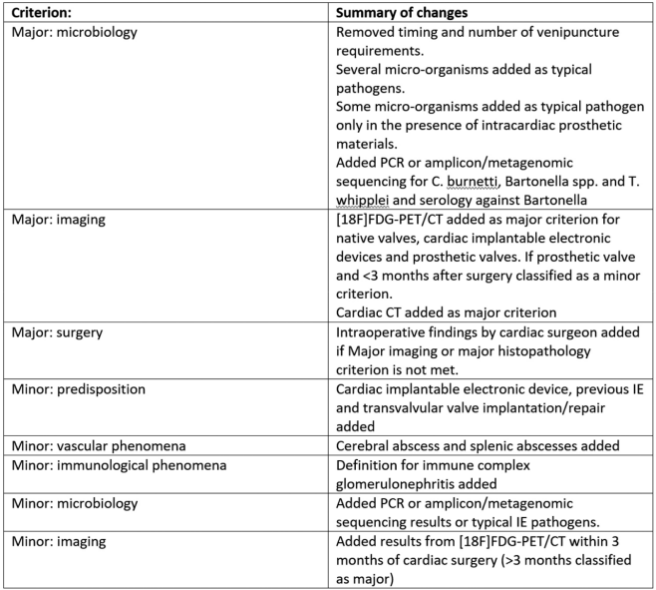
Overview of changes in criteria from 2000 modified Duke to 2023 Duke-ISCVID

Adapted from: Fowler et al, Clinical Infectious Diseases, 2023

**Methods:**

The study population consisted of consecutively enrolled patients suspected to have IE and discussed by the IE team of the Amsterdam University Medical Center (AMC) between October 2016 and March 2021. A panel of international experts on IE assigned a final diagnosis, which served as the reference standard. We compared the definite classification of the 2023 Duke-ISCVID criteria against this reference standard. We also evaluated accuracy of the new criteria, excluding data obtained from cardiac surgery and pathology (“Clinical Criteria”). Lastly, we quantified the added value of the proposed changes to the criteria, and compared the 2023 Duke-ISCVID to previous criteria using the McNemar test. We performed sensitivity analyses in patients who underwent cardiac surgery and patients who underwent TEE.

**Results:**

595 patients with suspected IE were included, of whom 399 (67%) were adjudicated as having IE. 111 patients (19%) had prosthetic valve IE, 48 (8%) had cardiac implantable electronic device IE. The 2023 Duke-ISCVID criteria were more sensitive than the modified Duke or ESC criteria (84.2 vs 74.9 vs 80%,respectively, p < 0.001 for ISCVID vs ESC criteria) without significant loss of specificity (Table 2). After excluding cardiac surgery/pathology results, the 2023 Duke-ISCVID Clinical Criteria also had significantly better sensitivity than the modified Duke and ESC criteria, again without losing specificity. The changes in the 'major microbiological' and 'imaging' criteria had the most impact (Figure 1). Findings were robust in sensitivity analyses (Table 3). When classifying ‘definite’ and ‘possible’ IE as a positive test, the 2023 Duke-ISCVID criteria had 99% sensitivity compared to the reference standard, but specificity decreased to 21%.

**Table 2:**
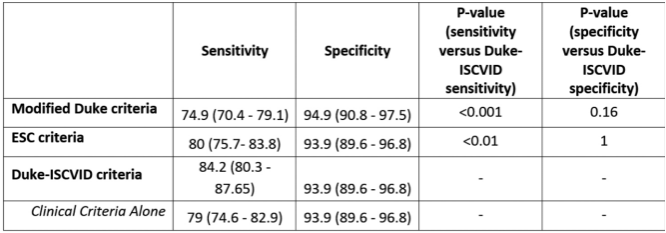
Diagnostic accuracy of modified Duke, ESC and Duke-ISCVID criteria

Sensitivity and specificity are against the reference standard (adjudication panel). The Clinical Criteria classification is the Duke-ISCVID classification when results from surgery or post-mortem studies are not used to determine classification.

**Table 3:**
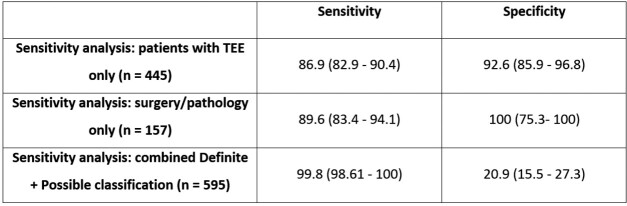
Sensitivity analyses of accuracy of Duke-ISCVID criteria

In the Definite + Possible analysis, patients with possible IE according to the Duke-ISCVID criteria were also considered as having IE. All comparisons are against the reference standard (adjudication panel).

**Conclusion:**

In this cohort, the 2023 Duke-ISCVID criteria represent a significant advance in the diagnostic classification of patients suspected of IE.

Added value of new Duke-ISCVID criteria compared to modified Duke criteria

**Figure:**
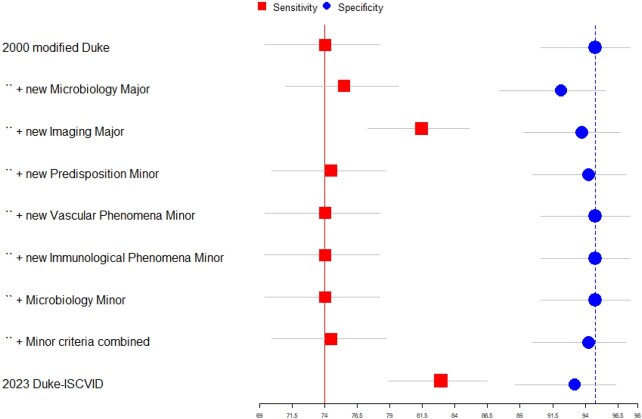

Each point (red square for sensitivity, blue circle for specificity) provides the diagnostic accuracy measure for the 2000 modified Duke Criteria with addition of a specific change from the Duke-ISCVID criteria. The horizontal bars represent the 95% confidence interval for the point estimate. The top points represent the modified Duke criteria without addition, the bottom points are the complete Duke-ISCVID criteria.

**Disclosures:**

**Larry M. Baddour, MD**, Boston Scientific: Advisor/Consultant|Roivant Sciences: Advisor/Consultant **Arnold S. Bayer, MD**, Akagera Medicines: Grant/Research Support|ContraFect Corporation: Grant/Research Support **Emanuele Durante Mangoni, MD,, PhD**, Advanz pharma: Advisor/Consultant|Advanz pharma: Grant/Research Support|Advanz pharma: Honoraria|Angelini: Grant/Research Support|Angelini: Honoraria|Genentech: Advisor/Consultant|Genentech: Board Member|Genentech: Honoraria|Infectopharm: Grant/Research Support|Menarini: Board Member|Menarini: Honoraria|Pfizer: Advisor/Consultant|Pfizer: Board Member|Pfizer: Grant/Research Support|Pfizer: Honoraria|Roche: Advisor/Consultant|Roche: Board Member|Roche: Honoraria **Thomas L. Holland, MD**, Aridis: Advisor/Consultant|Basilea Pharmaceutica: Advisor/Consultant|Karius: Advisor/Consultant|Lysovant: Advisor/Consultant **Adolf W. Karchmer, MD**, Karius: Grant/Research Support|Up To Date: Honoraria **Vance G. Fowler, MD, MHS**, Amphliphi Biosciences, Integrated Biotherapeutics; C3J, Armata, Valanbio; Akagera, Aridis, Roche, Astra Zeneca: Advisor/Consultant|Genentech, Regeneron, Deep Blue, Basilea, Janssen;: Grant/Research Support|Infectious Diseases Society of America: Honoraria|MedImmune, Allergan, Pfizer, Advanced Liquid Logics, Theravance, Novartis, Merck; Medical Biosurfaces; Locus; Affinergy; Contrafect; Karius;: Grant/Research Support|Novartis, Debiopharm, Genentech, Achaogen, Affinium, Medicines Co., MedImmune, Bayer, Basilea, Affinergy, Janssen, Contrafect, Regeneron, Destiny,: Advisor/Consultant|Sepsis diagnostic: Patent pending|UpToDate: Royalties|Valanbio and ArcBio: Stock Options

